# Data in support of proteomic and comparative genomic analysis reveal adaptability of *Brassica napus* to phosphorus-deficient stress

**DOI:** 10.1016/j.dib.2015.02.001

**Published:** 2015-02-12

**Authors:** Shuisen Chen, Guangda Ding, Zhenhua Wang, Hongmei Cai, Fangsen Xu

**Affiliations:** a National Key Laboratory of Crop Genetic Improvement, Huazhong Agricultural University, Wuhan 430070, China; b Microelement Research Center, Huazhong Agricultural University, Wuhan 430070, China

## Abstract

This data article contains data related to the research article titled proteomic and comparative genomic analysis reveal adaptability of *Brassica napus* to phosphorus-deficient stress [1]. Proteome alterations of roots and leaves in two *B. napus* contrasting genotypes, P-efficient ‘Eyou Changjia’ and P-inefficient ‘B104-2’, under long-term low phosphorus (P) and short-term P-free starvation was investigated, and then comparative gnomic analysis was conducted to interpret the interrelation of the differential abundance protein species responding to P deficiency with quantitative trait loci (QTLs) for P deficiency tolerance. The report concluded with the results that nearly 50% of the identified protein species was mapped in the confidence intervals of QTLs for P efficiency related traits. The tables presented here represented the detail information of protein spots detected, as well as protein species identified.

**Specifications table**

**Table:** 

Subject area	*Biology*
More specific subject area	*Plant proteomics*
Type of data	*Tables*
How data was acquired	***2-DE image analysis**: image analysis software PD-Quest 8.0 (Bio-Rad, USA)*
	***Mass spectrometry**: Matrix-assisted laser desorption/ionization-time of flight (Applied Biosystems, USA)*
Data format	*Processed*
Experimental factors	*No pretreatment of samples was performed*
Experimental features	*Total protein was extracted from roots and leaves of ‘Eyou Changjia’ and ‘B104-2’, respectively. 2-DE was performed to discover protein spots with abundance altered at least* ±2*-fold (T-test P<0.05).*
Data source location	*N/A*
Data accessibility	*Data is provided in Supplementary materials directly with this article*

**Value of the data**

• The data further validate the information presented in Chen et al. [1].
• The data provide the detail information of spots detection.
• The data provide the detail information of identified protein species.

## 1 Experimental design

Two *Brassica napus* genotype under two kinds of P treatments, long-term low P stress and short-term P-free starvation, were conducted, and three time points were used in each P treatment. Total protein was extracted from roots or leaves respectively of two *B. napus* by triplicate. 2-DE images were generated and compared to gain spots with abundance altered at least ±2-fold (*T*-test *p*<0.05). Then protein spots were identified by MALDI-TOF MS.

## 2 Materials and methods

### 2.1 Plant materials and growth conditions

P-efficient ‘Eyou Changjia’ and P-inefficient ‘B104-2’ that used in the present study were selected from 194 rapeseed (*B. napus*) cultivars by Duan et al. [2]. Seeds were surface sterilized with 10% (w/v) sodium hypochlorite for 5 min and then washed 3 times in deionized water (dH_2_O). The surface-sterilized seeds were germinated on moistened gauze until root length about 2 cm. For long-term low P stress experiment, half of the seedlings were grown in a nutrient solution containing 1.4 µM Na_2_HPO_4_ and 3.6 µM NaH_2_PO_4_ (LP, 5 µM P) for 18 days after transplanting, then the seedlings were shifted to nutrient solution containing 36 µM Na_2_HPO_4_ and 144 µM NaH_2_PO_4_ (HP, 200 µM P) for additional 2 to 5 days. The remaining seedlings were grown in HP solution, which was used as the control. In addition to the P, the basal complete nutrient solution contained: 0.24 g/L NH_4_NO_3_, 0.50 g/L MgSO_4_, 0.15 g/L KCl, 0.36 g/L CaCl_2_, 0.05 mM EDTA-Fe and Arnon microelement solution [3]. Roots and leaves of both genotypes were harvested separately on the 18th, 20th and 23rd day after transplanting, which were marked as 18, 18+R2 and 18+R5, respectively. For short-term P-free starvation, all of the seedlings were grown under +P (200 µM P) for 15 days, then half the seedlings were shifted to the P-free solution (−P, 0 µM P) and the remaining seedlings were maintained under +P conditions as the control. The roots and leaves were harvested at 0, 1, 3 and 5 days after the P was removed. For proteomic analysis, the 1st and 2nd euphylla next to the cotyledon from three seedlings were collected as one leaf sample, and the corresponding three roots of the seedling were collected as one root sample. Each sample was replicated biologically three times (Supplementary Fig. S3). Seedlings were grown in an illuminated culture room (300–320 μmol/m^2^/s, 24 °C day/22 °C night, 16 h photoperiod). The nutrient solution was refreshed every 5 days, which was supplied initially with 1/4 full-strength nutrient solution, then 1/2 and full-strength in turn. After the fresh weights were measured, both root and leaf samples were immediately chilled in liquid nitrogen and stored at −80 °C for further using.

### 2.2 Extraction and quantification of total protein

The root protein was extracted as described in our previous study [4]. And the leaf protein was extracted using TCA/acetone method as described by Wang et al. [5]. The proteins (control and treated) were extracted from three independent biological replicates, respectively. Then each biological replicate was used as an independent sample for protein content determination using Bradford method [6] with series of concentration gradient of BSA as a standard before 2-DE, in which 2 µL and 4 µL of the extracted protein solutions were used, respectively. And the protein yield was calculated for each sample (Table S2).

### 2.3 2-DE and images analysis

For 2-DE, 17 cm IPG strip (Bio-Rad, USA) with liner gradient pH range (pH 5–8) was selected. For each strip 1000 µg root protein or 1500 µg leaf protein extracts were loaded to each IPG strip in first dimension, and then 12% polyacrylamide gels were used in the second dimension as previously described [4]. The gels were stained by coomassie brilliant blue and scanned using a GS-800 densitometer (Bio-Rad, USA), then the image analysis software PDQuest 8.0 (Bio-Rad, USA) were used for spots detecting. Local regression method (LOESS) normalization was selected to correct the differences between the gels. Spots abundance showing at least two-fold alteration and the *P*<0.05 based on Student’s *T*-test were considered as DAPs. Qualitative difference and quantitative differences were showed in Supplementary Table S2.

### 2.4 MALDI-TOF/TOF MS and protein identification

The selected spots were manually excised from the gels. After alkylated and reduced, the trypsin-digested protein spots were automatically transferred to MALDI-TOF/TOF analyzer (Applied Biosystems, USA). Both the MS and MS/MS data were submitted to Mascot (Version 2.2, Mtrix Science Ltd, London, UK) for protein species identification. The search results were evaluated by protein score confidence interval (C.I.%) calculated in GPS Explorer software (Applied Biosystems), which is based on the MASCOT score. Only those identified protein species with a C.I.%>99% were accepted (Table S1) [1].
